# Relationship of oxidized low density lipoprotein with lipid profile and oxidative stress markers in healthy young adults: a translational study

**DOI:** 10.1186/1476-511X-10-61

**Published:** 2011-04-19

**Authors:** Kiriaque BF Barbosa, Ana Carolina P Volp, Helen Hermana M Hermsdorff, Iñigo Navarro-Blasco, M Ángeles Zulet, J Alfredo Martínez, Josefina Bressan

**Affiliations:** 1 Nutrition Center, Federal University of Sergipe, Aracaju, Brazil; 2 Department of Clinical and Social Nutrition, Federal University of Ouro Preto, Ouro Preto, Brazil; 3 Department of Nutrition, Food Science, Physiology and Toxicology, University of Navarra, Pamplona, Spain; 4 Department of Chemistry and Soil Science, Universidad de Navarra, Pamplona, Spain; 5 Department of Nutrition and Health, Federal University of Viçosa, Viçosa, Brazil

## Abstract

**Background:**

Despite oxidized low density lipoprotein (ox-LDL) plays important roles in the pro-inflammatory and atherosclerotic processes, the relationships with metabolic and oxidative stress biomarkers have been only scarcely investigated in young adult people. Thus, the aim of this study was to assess plasma ox-LDL concentrations and the potential association with oxidative stress markers as well as with anthropometric and metabolic features in healthy young adults.

**Methods:**

This study enrolled 160 healthy subjects (92 women/68 men; 23 ± 4 y; 22.0 ± 2.9 kg/m^2^). Anthropometry, body composition, blood pressure, lifestyle features, biochemical data, and oxidative stress markers were assessed with validated tools. Selenium, copper, and zinc nail concentrations were measured by atomic absorption spectrophotometry.

**Results:**

Total cholesterol (TC), LDL-c and uric acid concentrations, TC-to-HDL-c ratio, and glutathione peroxidase (GPx) activity were positive predictors of ox-LDL concentrations, while nail selenium level (NSL) was a negative predictor, independently of gender, age, smoking status, physical activity. Those individuals included in the highest tertile of GPx activity (≥611 nmol/[mL/min]) and of NSL (≥430 ng/g of nail) had higher and lower ox-LDL concentrations, respectively, independently of the same covariates plus truncal fat or body mass index, and total cholesterol or LDL-c concentrations.

**Conclusions:**

Ox-LDL concentrations were significantly associated with lipid biomarkers, GPx activity, uric acid concentration, and NSL, independently of different assayed covariates, in young healthy adults. These findings jointly suggest the early and complex relationship between lipid profile and redox status balance.

## Background

A lipid profile characterized by reduced high density lipoprotein-cholesterol (HDL-c) concentrations and increased low density lipoprotein-cholesterol (LDL-c) and triglycerides concentrations as well as increased total cholesterol-to-HDL-c ratio constitutes a high risk for type 2 diabetes, metabolic syndrome and cardiovascular diseases [1-3]. In turn, oxidative stress impairment or altered antioxidant status have been suggested as pivotal keys in the onset of certain chronic diseases [4,5].

In this sense, oxidized low-density lipoproteins (ox-LDL), a recognized oxidative stress marker, has been positively associated with central obesity [6], metabolic syndrome manifestation [7] and atherosclerosis [8]. Also, uric acid has been proposed as independent risk factor for cardiovascular diseases [9,10], in addition to implication in LDL-c oxidation and generation of an oxidative status in hyperuricemia conditions [11,12]. In turn, glutathione peroxidase is an enzyme with relevant antioxidant role in the redox balance [4], while selenium is an essential mineral, which have been investigated by its antioxidant and anti-inflammatory proprieties in the preventing chronic disorders [13-15]. However, the relationship of ox-LDL with lipid and oxidative stress biomarkers has been only modestly investigated in young adult people [16], while its association with nail selenium levels has not been apparently reported.

Overall, this study aimed to assess plasma ox-LDL concentrations and the potential associations with oxidative stress markers as well as with anthropometric and metabolic (glucose and lipid profiles) data in healthy young adults. Thus, we measured plasma uric acid concentrations and glutathione peroxidase (GPx) activity in erythrocytes, since an altered regulation of these markers has been associated with ox-LDL concentrations in oxidative stress and chronic disorders conditions [11,12,17,18]. Also, we also assessed nail levels of three trace elements related to antioxidant defense mechanisms (selenium, zinc and cooper), whose levels have presented relevant associations with biomarkers in young adults [13,14,19].

## Subjects and methods

### Subjects

In this study participated 160 subjects (92 women and 68 men) with a mean age of 23 ± 4 years old (range 18-35) and a mean body mass index (BMI) of 22.0 ± 2.9 kg/m^2 ^(range 18.5-34.9). Exclusion criteria were any diagnosed organic underlying disease (gastrointestinal, kidney, liver, respiratory or heart disease), cancer, infectious and inflammatory disorders, diabetes (fasting glucose level > 126 mg/dl), hypertension (systolic and diastolic blood pressure values ≥ 140 and 90 mmHg, respectively), pregnancy, disorders affecting body composition (e.g. lipodystrophy and Cushing syndrome) or lipid-lowering treatment. Other exclusion criteria were contraceptive use up to 2 months before participation in this study, recent follow up of diets designed for weight loss or unstable weight (change > 10% in habitual weight) in the past 6 months. In accordance with the principals of the Helsinki Declaration and after a clear explanation of the study protocol, each participant gave a written informed consent to participate. The study was approved by the Committee of Ethics in Research with Human Beings of the Federal University of Viçosa (Of. Ref. n° 009/2006).

### Anthropometric and body composition assessments

Anthropometric and body composition were determined in all the subjects after 12 h of fasting. Body weight was measured with an electronic microdigital scale balance (Tanita TBF-300A model, Tokyo, Japan) to the nearest 0.1 kg, while height was assessed with a stadiometer (Seca 206 model, Hamburg, Germany) to the nearest 0.1 cm. Thus, BMI was calculated by the ratio between weight (kg) and the squared height (m^2^), which was applied to categorize normal-weight (18.5-24.9 kg/m^2^), overweight (25-29.9 kg/m^2^), and obese (BMI ≥30 kg/m^2^) subjects, according to the World Health Organization criteria [20]. Waist circumference was measured midway between the lowest rib and the iliac crest and hip circumference was determined at the maximal hip circumference without gluteus contraction [21], both with an inelastic and flexible tape to the nearest 0.1 cm. The waist-to-hip ratio was also calculated. Triceps, biceps, subscapular and suprailiac skinfold thicknesses (ST) were measured to the nearest 1 mm by using a skinfold caliper (Lange caliper, Cambridge Scientific Industries Inc., Cambridge Maryland, USA), according to a previously described protocol [22]. The sum of STs (mm) was also calculated. Truncal fat was also calculated as the sum of subscapular and suprailiac ST divided by the sum of 4 ST, expressed in percentage [13,23]. Total body fat (%, to the nearest 0.1%) and body fat mass (kg) were measured by an impedance bioelectric device (Biodynamics 310 model, Washington, USA).

### Blood pressure and biological sample measurements

Systolic and diastolic blood pressure values were measured twice by a mercury sphygmomanometer (BIC, São Paulo, Brazil) to the nearest 2 mmHg as described elsewhere [24].

Blood samples were draw by vein puncture after a 12 h overnight fast. The plasma and serum samples were separated from whole blood by centrifugation at 2465 *g *× 15 min at 5°C (Eppendorf AG, 5804R model, Hamburg, Germany) and were immediately stored at -80°C until assay. Serum glucose, total cholesterol, HDL-c, triacylglycerols, and uric acid concentrations were assessed by specific colorimetric assays (Bioclin, Quibasa, Minas Gerais, Brazil), using an automated analyzer system (BS-200, Shenzhen Mindray Bio-medical Electronics Co., Nanshan, China). LDL-c data were calculated by the Friedewald equation as described elsewhere [25]. The total cholesterol-to-HDL-c ratio was also calculated [26]. Plasma insulin concentrations (sensitivity 2 μU/mL) were measured by an ELISA assay kit (Linco Research, St. Charles, USA). Insulin resistance was estimated by the homeostasis model assessment of insulin resistance (HOMA-IR) calculated as fasting glucose (mmol/L) × fasting insulin (μU/mL)/22.5 [27]. Plasma ox-LDL concentrations (sensitivity < 6.56 U/L) were measured by an ELISA assay kit (Mercodia, Uppsala, Sweden). GPx activity (nmol/[mL/min]) was measured in erythrocytes by a commercially available kit (Cayman Chemical, Ann Arbor, USA). Of total sample, 135 participants delivered fingernail and toenail samples as requested. Fingernail and toenail samples were treated with sub-boiling nitric acid in a high-pressure teflon digestion vessel using a microwave digestion system (Ethos Plus, Millestone, Sorisole, Italy). Selenium (ng/g of nail), copper and zinc (μg/g of nail) concentrations were measured by a Perkin Elmer Analyst 800 atomic absorption spectrometer (Norwalk, CT, USA) as previously described [28,29].

### Other lifestyle measurements

As covariates, lifestyle features were also determined. Thus, the participants were asked about smoking status (never, former, or current smokers) and, if it was the case, how many cigarettes they smoked per day. Also, they were requested whether some vitamin supplement was consumed (Yes/No). In respect to physical activity, the participants declared about to regular physical activity practice (Yes/No), and if it was the case, the type and the volume of activity (h/week). To quantify the volume of activity, a metabolic equivalent (MET) index was also computed by assigning a multiple of resting metabolic rate (MET score) to each activity [30], followed by the sum over all activities to obtain a value of overall weekly MET/h as described elsewhere [31].

### Statistical analysis

Results are shown as mean ± SD. The Kolmogorov-Smirnov normality test was used to determine variable distribution. In order to detect difference in anthropometrical, lifestyle, metabolic and oxidative stress data in respect to ox-LDL concentrations, this was taken as suitable variable considering its median as cutoff value (69.4 U/L) and categorizing the subsequent population group in "low" and "high" ox-LDL concentrations according to this value (< or ≥ 69.4 U/L, respectively). The median cutoff criteria have been previously applied [13,19] and is based on a valid and reliable method to assign two groups of risk in epidemiological studies [32]. Accordingly, statistical comparisons between groups were performed by the parametric Student *t*-test, Manny-Whitney *U*-test or chi-square (χ^2^) test as appropriate. The Spearman correlation coefficients were used to screen the potential associations between ox-LDL concentrations and interest variables. In addition, multivariate linear regression models were also applied to further explain the associations of ox-LDL concentrations with these variables. The linear regression models were adjusted for gender, age, smoking status, physical activity practice. Confidence intervals (95% CIs) were used to describe linear regression coefficient (β).

We also categorized the participants by tertiles of GPx activity, nail selenium levels, and uric acid concentrations, since these biomarkers were significantly associated with ox-LDL in the linear regression analyses previously described in this section. Linear trends were assessed by assigning the median value to each tertile of these variables and modeling these values as a continuous variable. Subsequently, we performed linear regression analyses, including ox-LDL as dependent variable, tertiles of GPx activity, nail selenium levels, and uric acid concentrations as independent variables, and gender, age, smoking status, physical activity practice, total cholesterol or LDL-c concentrations, truncal fat or BMI, and uric acid (in some cases) as control covariates. Since the GPx activity and selenium nail levels were measured in a fewer number of participants (n = 100 and n = 135, respectively), we tested *a posteriori *the statistical power (1-β) to trends of these markers with ox-LDL, using effect size f^2 ^(based in corrected R^2 ^values), *P*-value from model < 0.05, total sample size and the number of independent predictors as input parameters, in the G*Power version 3.0.10. A *P-*value < 0.05 was considered statistically significant, and the statistical analyses were performed using SAS system 8.0 (SAS Institute Inc., Cary, USA).

## Results

Anthropometric, clinical and biochemical data (mean ± SD) categorized by the median value of plasma ox-LDL concentrations are reported in Table 1. Individuals with high concentrations of ox-LDL (≥69.4 U/L) showed significantly higher values of BMI, total cholesterol, LDL-c, total cholesterol-to-HDL-c ratio, uric acid, and GPx activity, while nail selenium levels were significantly lower. Gender distribution, anthropometric and body composition measurements, except BMI, glucose profile, and blood pressure did not differ, when classified by ox-LDL concentrations. No differences were found concerning to lifestyle features, when categorized by the median value of ox-LDL concentrations (Table 2).

**Table 1:** Anthropometric, clinical, and biochemical data, categorized by the median (cutoff: 69.4 U/L) of ox-LDL concentrations (n = 160)

	Low ox-LDL <69.4 U/L (n = 80)	High ox-LDL ≥ 69.4 U/L (n = 80)	*P*-value*
Women (%)	60.8	52.6	0.305
Age (y)	23.1 ± 3.6	23.4 ± 3.4	0.145
BMI (kg/m^2^)	21.6 ± 2.8	22.4 ± 3.0	*0.046*
Waist circumference (cm)	78.0 ± 8.7	78.3 ± 8.7	0.863
Waist-to-hip ratio	0.8 ± 0.1	0.8 ± 0.1	0.469
Sum of 4 ST (mm)	42.6 ± 16.8	45.0 ± 20.4	0.369
Truncal fat (%)	57.4 ± 7.1	59.3 ± 6.1	0.069
Total body fat (%)	23.8 ± 6.1	23.4 ± 7.0	0.735
Body fat mass (kg)	15.0 ± 5.2	14.7 ± 5.4	0.303
Systolic blood pressure (mmHg)	109 ± 9	110 ± 9	0.239
Diastolic blood pressure (mmHg)	74 ± 7	73 ± 7	0.257
Glucose (mg/dL)	90.8 ± 7.0	90.3 ± 6.3	0.394
Insulin (μU/mL)**	10.3 ± 5.0	10.2 ± 6.0	0.302
HOMA-IR**	2.3 ± 1.2	2.3 ± 1.3	0.245
Total cholesterol (mg/dL)	153.4 ± 27.8	166.5 ± 33.5	*0.007*
HDL-c (mg/dL) ^†^	47.3 ± 12.4	45.7 ± 10.4	0.342
LDL-c (mg/dL)	91.0 ± 26.6	100.2 ± 26.4	*0.009*
Triacylglycerol (mg/dL)	96.1 ± 49.1	103.2 ± 38.5	0.059
Total cholesterol-to-HDL-c ratio^†^	3.3 ± 0.6	3.8 ± 0.9	*<0.001*
Uric acid (mg/dL)	3.4 ± 1.1	3.7 ± 1.1	*0.049*
GPx activity (nmol/[mL/min])^‡^	487.9 ± 231.3	659.1 ± 299.2	*0.002*
Selenium (ng/g of nail)^§^	396.2 ± 88.0	365.5 ± 76.7	*0.033*
Zinc (μg/g of nail)^§^	124.5 ± 57.7	132.3 ± 68.7	0.212
Copper (μg/g of nail)^§^	7.4 ± 5.5	7.1 ± 7.1	0.247

Data are mean ± SD.

Ox-LDL, oxidized low density lipoprotein; BMI, body mass index; ST, skinfold thickness; HOMA-IR, homeostasis model assessment of insulin resistance; HDL-c, high-density lipoprotein cholesterol; LDL-c, low-density lipoprotein cholesterol; GPx, glutathione peroxidase.

*Student *t *test was performed for variables with normal distribution while remaining variables were analyzed by Mann-Whitney *U *test, as appropriate.

**n = 79 and n = 77, for low and high ox-LDL, respectively.

^†^n = 73 for high ox-LDL.

^‡^n = 44 and n = 56, for low and high ox-LDL, respectively.

^§ ^n = 69 and n = 66, for low and high ox-LDL, respectively.

**Table 2:** Lifestyle features of the participants, categorized by the median (cutoff: 69.4 U/L) of ox-LDL concentrations*

*Lifestyle features*	Low ox-LDL <69.4 U/L (n = 80)	High ox-LDL ≥ 69.4 U/L (n = 80)	***P*- value**^**†**^
Vitamin supplement use (%)	6.2	6.2	0.721
Smokers (%)^‡^	9.6	13.4	0.105
Smoking (cigarettes/d)^‡^	1.3 ± 4.9	1.6 ± 4.9	0.318
Self-reported PA practice (%)^‡^	72.6	70.1	0.116
MET (h/wk)^‡^	116 ± 105	135 ± 116	0.324

Ox-LDL, oxidized low density lipoprotein; PA, physical activity; MET, activity metabolic equivalent

*Data are mean ± SD or frequencies.

^†^*P*-value from *χ*^*2 *^test and Mann-Whitney *U *test for dichotomous and continuous variables, respectively.

^‡^n = 73 and n = 67, for low and high ox-LDL, respectively.

To better understand the associations between ox-LDL concentrations and some variables of interest, Sperman's coefficient correlations were performed. Although all anthropometric variables presented positive trends with higher ox-LDL values (see Additional file 1: Table S1), only truncal fat significantly correlated with ox-LDL concentration (r_S _= 0.16, *P *= 0.043). Regarding biochemical data and antioxidant defense system components, the following statistical correlations were detected: total cholesterol (r_S _= 0.23, *P *= 0.003), LDL-c (r_S _= 0.22, *P *= 0.004), triacylglicerol (r_S _= 0.013, *P *= 0.013), total cholesterol-to-HDL-c ratio (r_S _= 0.41, P < 0.001), nail selenium (r_S _= - 0.19, *P *= 0.026) and copper (r_S _= - 0.17, *P *= 0.046) concentrations, and GPx activity (r_S _= 0.29, *P *= 0.003). In addition, systolic blood pressure significantly correlated with ox-LDL concentration (r_S_= 0.17, *P *= 0.027).

In Table 3, linear regression analysis showed that lipid biomarkers, such as total cholesterol, LDL-c, total cholesterol-to-HDL-c ratio as well as uric acid concentrations and GPx activity were positive predictors of circulating concentrations of ox-LDL, after adjusted for sex, age, smoking status, and physical activity. In turn, nail selenium level was a negative predictive factor of ox-LDL levels. The increase of 1 unit on nail selenium concentration (1 ng/g of nail) was associated with a reduction of 0.06 U/L in ox-LDL circulating levels. The nail copper concentration had a similar effect, but no statistical significance was found.

**Table 3:** Multivariate linear regression analysis with ox-LDL concentrations (U/L) as a dependent variable (n = 160)*

Predictors of ox-LDL	β coefficient (95% CI)	*P*	**R**^**2**^
BMI (kg/m^2^)	1.206 (-0.347 to 2.760)	0.127	0.008
Truncal fat (%)	0.489 (-0.193 to 1.172)	0.158	0.006
Systolic blood pressure (mmHg)	4.749 (-0.127 to 9.626)	0.056	0.016
Total cholesterol (mg/dL)	0.228 (0.086 to 0.370)	*0.001*	*0.054*
LDL-c (mg/dL)	0.216 (0.048 to 0.384)	*0.012*	*0.033*
Triacylglycerol (mg/dL)	0.082 (-0.021 to 0.185)	0.118	0.009
Total cholesterol-to-HDL-c ratio**	15.787 (10.776 to 20.798)	*<0.001*	*0.198*
Uric acid (mg/dL)	4.465 (0.342 to 8.588)	*0.034*	*0.022*
GPx activity (nmol/[mL/min])^†^	0.029 (0.008 to 0.050)	*0.007*	*0.062*
Selenium (ng/g of nail)^§^	-0.063 (-0.119 to -0.007)	*0.025*	*0.029*
Copper (μg/g of nail)^§^	-0.573 (-1.322 to 0.176)	0.132	0.009

Ox-LDL, oxidized low density lipoprotein; BMI, body mass index; ST, skinfold thickness; LDL-c, low-density lipoprotein cholesterol; HDL-c, high-density lipoprotein cholesterol,

*Multivariate linear regressions, adjusted for gender, age, smoking, and physical activity.

**n = 153, ^† ^n = 100, ^§^n = 135.

**Figure 1:**
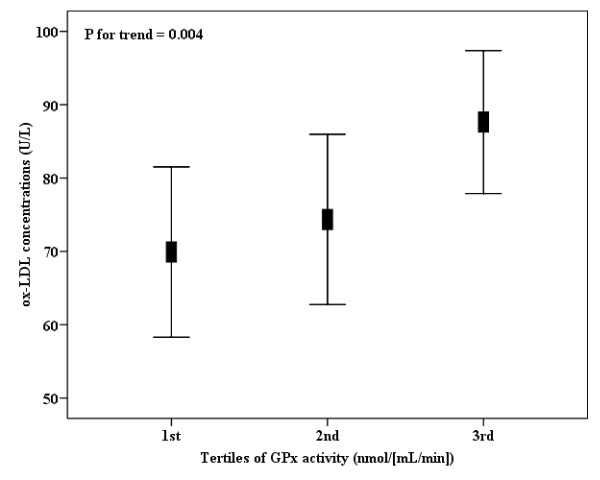
**Plasma ox-LDL concentrations (n = 100), according to tertiles of GPx activity**. GPx activity into tertiles, 1st: <334, n = 33; 2nd: 334-611, n = 34; 3rd: ≥611 nmol/[mL/min], n = 33. Data are means and 95% CIs. *P *for trend, from linear regression models adjusted for gender, age, smoking status, physical activity, truncal fat, uric acid and total cholesterol concentrations.

**Figure 2:**
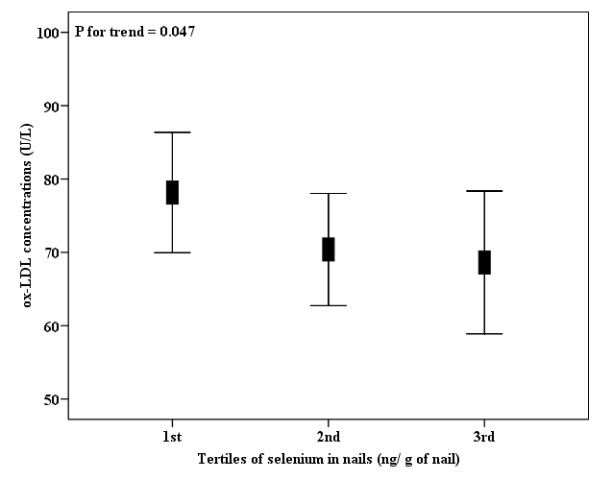
**Plasma ox-LDL concentrations (n = 135), according to tertiles of nail selenium levels**. Nail selenium levels into tertiles, 1st: <330, n = 45; 2nd: 330-430, n = 45; 3rd: ≥430 ng/g of nail, n = 45. Data are means and 95% CIs. *P *for trend, from linear regression models adjusted for gender, age, smoking status, physical activity, truncal fat, uric acid and total cholesterol concentrations.

Interestingly, ox-LDL concentrations were higher in the subjects included in the higher tertile of GPx activity (Figure 1), independent from gender, age, smoking status, physical activity, truncal fat, uric acid, and total cholesterol concentrations. Also, ox-LDL concentrations were statistically decreased across tertiles of nail selenium values, independent from the same confounding factors (Figure 2). When truncal fat was substituted by BMI and total cholesterol was substituted by LDL-c concentration in linear regression models, the same trend and statistical outcomes were found concerning tertiles of GPx activity or nail selenium levels (data not shown). Since we measured the GPx activity and selenium nail levels in fewer number of participants (n = 100 and n = 135, respectively), we *a posteriori *tested the statistical power of the analyses, which was satisfactory for both outcomes (1-β = 0.81 and 1-β = 0.96, respectively).

Finally, the trend of ox-LDL concentrations into tertiles of uric acid was also tested in linear regression model adjusted for gender, age, smoking status, physical activity, truncal fat, and total cholesterol concentrations. Despite the trend was positive, it did not achieve statistical significance (*P *> 0.05).

## Discussion

In this cross-sectional study, ox-LDL concentrations were positively associated with GPx activity and inversely associated with nail selenium levels, both recognized antioxidant markers, in healthy young adults.

GPx is an important antioxidant enzyme, which has been used as oxidative stress marker concerning to an altered antioxidant balance [4,33]. Previous studies have supported the positive predictive effect of GPx activity on circulating levels of ox-LDL [16,18]. Experimental studies showed an increase in the activity of this enzyme in endothelial cells or macrophages treated with ox-LDL, as a protective mechanism against the increased generation of reactive oxygen species induced by ox-LDL [17,18], while other observational study reported also a positive association between GPx activity and ox-LDL in healthy young Spanish adults, despite it was not statistically significant [16]. Thus, our findings are in agreement to the hypothesis that a positive association between GPx activity and ox-LDL might constitute a consequence of the high ox-LDL concentrations, being an adaptive mechanism to prevent further oxidative imbalance.

In turn, our study demonstrated, apparently for the first time, the negative association of nail selenium levels and ox-LDL concentrations, whereas an increase of 1 ng of selenium per g of nail was associated with a decrease of 0.06 U/L in ox-LDL. Selenium is an essential antioxidant mineral, whereas its increased consumption has been inversely associated with pro-inflammatory markers [34,35] as well as with lower hypercholesterolemia [36,37] and lower LDL susceptibility to oxidation [38]. In addition, increased nail selenium levels also have been related to lower pro-inflammatory marker concentrations, such as complement C3 factor, asymmetric dimethylarginine, and interleukin-18 in young healthy adults [13,14,19]. According to previous and onset findings, it could speculate an inverse relationship between dietary selenium intake and the oxidation of LDL-c. Moreover, our finding reinforces the measurement of this mineral in the nail as a promising alternative to assess the relationship of dietary selenium and pro-inflammatory and oxidative stress markers, since it is a good indicator of dietary selenium intake [39], which in turn, has a limited assessment by the scarcity of information in the tables of food composition and by influence of several factor on its bioavailability [40].

Furthermore, ox-LDL was positively associated with other lipid biomarkers, such as total cholesterol and LDL-c, in accordance to previous studies [7,8,16,41,42]. Interestingly, the participants of this study are young adults predominantly normolipidemic (total cholesterol <200 mg/dL and LDL-c <160 mg/dL represent 92.5 and 97.5% of the sample, respectively). Thus, it is noteworthy that the increase of 1 mg/dL in serum total cholesterol or in LDL-c as well as of one unit in the total cholesterol-to-HDL-c ratio was predictors of an increment of 0.22; 12.21 and 15.78 U/L in ox-LDL concentrations, respectively. Thus, despite the cross-sectional nature of this study, we could speculate that the positive association between lipid profile and ox-LDL - a recognized oxidative stress marker - occurs early and could explain, at least in the part, the time-course dependent relationships between oxidative stress and chronic disorders in middle-aged and older subjects.

Other relevant outcome of this study was the relationship between uric acid and ox-LDL concentrations, independently of gender, age, smoking status, physical activity, whereas the addition of 1 mg/dL in serum uric acid was associated with the increase of plasma ox-LDL in 4.4 U/L. In this sense, hyperuricemia (≥ 7 mg/dL) has been considered a risk factor for cardiovascular diseases [9,10,43] and a positive predictor of the occurrence of small and dense LDL-c, more susceptible to oxidation [12]. Moreover, uric acid concentration higher than 4 mg/dL appears to have a pro-oxidant redox effect [11], in addition to its synthesis can lead to the generation of superoxide anion radicals, hydroxyl and hydrogen peroxide [44]. The results reported by other authors suggest the role of uric acid in the relationship between oxidative stress and cardiovascular diseases, while the finding of this study might establish a new link of uric acid with oxidative conditions. However, the association between uric acid and ox-LDL was attenuated after adjusting for truncal fat and cholesterol total concentrations, indicating that this relation could be conditioned by other oxidative and metabolic-related risk factors, as previously postulated by other authors [11,43].

Regarding the association of ox-LDL concentrations with anthropometric and body fat distribution data, BMI was significantly higher in those individuals with high ox-LDL, while truncal was significantly positively associated with ox-LDL concentrations. However, both variables were not able to predict to ox-LDL concentrations, which is not in agreement with other studies [6-8]. In fact, the body fat distribution, characterized by central fat accumulation, has been associated with increasing in pro-inflammatory and oxidative stress markers [6,23,45]. In this context, the lack of associations between concentrations of ox-LDL and adiposity indicators in this study could be explained by the predominance of normal-weight individuals (BMI <25.0 kg/m^2^; 85% of the sample) or by relatively small size of sample.

Moreover, ox-LDL was not related to glucose biomarkers in young adults. On one hand, some studies have demonstrated the association of hyperglycemia and hyperinsulinemia with increased circulating levels of ox-LDL [7,41,46]. On the other hand, other authors found no significant correlations between circulating levels of ox-LDL and glucose biomarkers [42,47]. Likely, differences in the study sample, such as gender distribution, age, obesity degree or body fat distribution, might influence the outcomes [4].

Our study had certain limitations. The cross-sectional design did not clearly elucidate the cause-and-effect on the results. In addition, the residual confounders that may affect the oxidization of lipoproteins, but were not included in our present study (i.e. dietary factors), should also be considered. Finally, further replication in independent and larger samples would be convenient for a future translational application at a population level, although the sample size is adequate from the standpoint of the initial association discovery, with a satisfactory statistical power in the most relevant analyses of this work.

## Conclusions

In summary, ox-LDL concentrations were positively associated with specific lipid biomarkers (total cholesterol, LDL-c and total cholesterol-to-HDL-c ratio), GPx activity and uric acid concentration, and inversely associated with nail selenium levels, independent of different covariates, in young healthy adults. These findings jointly suggest the early and complex relationships between lipid profile and redox status balance, measured through oxidative and antioxidant markers.

## List of abbreviations

BMI: body mass index; GPx: glutathione peroxidase; HDL-c: high density lipoprotein-cholesterol; LDL-c: low density lipoprotein-cholesterol; HOMA-IR: homeostasis model assessment of insulin resistance; MET: metabolic equivalents; ox-LDL: oxidized low density lipoprotein; ST: skinfold thicknesses.

## Competing interests

The authors declare that they have no competing interests.

## Authors' contributions

KBFB: design, field work, data collection, analysis, and writing of the manuscript. ACPV and HHMH: design, field work, data collection and analysis. INB: analysis and financial management. MAZ: project co-leader, design, data interpretation, and editing the manuscript. JAM: project co-leader, design, financial management, data interpretation, and editing of the manuscript. JB: project leader, general coordination, design, financial management, data interpretation, and editing of the manuscript. All authors read and approved the final manuscript.

## Supplementary Material

Additional file 1 Table S1: Spearman bivariate correlation between anthropometric data and ox-LDL concentrations (n = 160). Click here for file

## References

[B1] Ingelsson E Schaefer EJ Contois JH McNamara JR Sullivan L Keyes MJ Pencina MJ Schoonmaker C Wilson PW D'Agostino RB Vasan RS Clinical utility of different lipid measures for prediction of coronary heart disease in men and women JAMA 2007 298 776 785 10.1001/jama.298.7.776 17699011

[B2] NCEP-ATPIII Executive Summary of The Third Report of The National Cholesterol Education Program (NCEP) Expert Panel on Detection, Evaluation, And Treatment of High Blood Cholesterol In Adults (Adult Treatment Panel III) JAMA 2001 285 2486 2497 10.1001/jama.285.19.2486 11368702

[B3] Hadaegh F Hatami M Tohidi M Sarbakhsh P Saadat N Azizi F Lipid ratios and appropriate cut off values for prediction of diabetes: a cohort of Iranian men and women Lipids Health Dis 2010 9 85 10.1186/1476-511X-9-85 20712907 PMC2933665

[B4] Pérez-Matute P Zulet MÁ Martínez JA Reactive species and diabetes: counteracting oxidative stress to improve health Curr Opin Pharmacol 2009 9 771 779 10.1016/j.coph.2009.08.005 19766058

[B5] Valdecantos MP Perez-Matute P Martinez JA Obesity and oxidative stress: role of antioxidant supplementation Rev Invest Clin 2009 61 127 139 19637727

[B6] Weinbrenner T Schroder H Escurriol V Fito M Elosua R Vila J Marrugat J Covas MI Circulating oxidized LDL is associated with increased waist circumference independent of body mass index in men and women Am J Clin Nutr 2006 83 30 35 quiz 181-182 16400046 10.1093/ajcn/83.1.30

[B7] Holvoet P Lee DH Steffes M Gross M Jacobs DR Jr Association between circulating oxidized low-density lipoprotein and incidence of the metabolic syndrome JAMA 2008 299 2287 2293 10.1001/jama.299.19.2287 18492970 PMC2562739

[B8] Santiago F Moriel P Correa R Nakamura R Gidlund M Schreiber R Barros-Mazon S Cotta de Faria E Atherosclerotic and metabolic repercussions of increased plasma levels of oxidized ldl and antibodies against oxidized ldl in asymptomatic adults [Abstract] Atherosclerosis 2010 11 P350 91

[B9] Fang J Alderman MH Serum uric acid and cardiovascular mortality the NHANES I epidemiologic follow-up study, 1971-1992. National Health and Nutrition Examination Survey JAMA 2000 283 2404 2410 10.1001/jama.283.18.2404 10815083

[B10] Niskanen LK Laaksonen DE Nyyssonen K Alfthan G Lakka HM Lakka TA Salonen JT Uric acid level as a risk factor for cardiovascular and all-cause mortality in middle-aged men: a prospective cohort study Arch Intern Med 2004 164 1546 1551 10.1001/archinte.164.14.1546 15277287

[B11] Hayden MR Tyagi SC Uric acid: A new look at an old risk marker for cardiovascular disease, metabolic syndrome, and type 2 diabetes mellitus: The urate redox shuttle Nutr Metab (Lond) 2004 1 10 10.1186/1743-7075-1-10 PMC529248 15507132

[B12] Vekic J Jelic-Ivanovic Z Spasojevic-Kalimanovska V Memon L Zeljkovic A Bogavac-Stanojevic N Spasic S High serum uric acid and low-grade inflammation are associated with smaller LDL and HDL particles Atherosclerosis 2009 203 236 242 10.1016/j.atherosclerosis.2008.05.047 18603253

[B13] Puchau B Zulet MA González de Echavarri A Navarro-Blasco I Martínez JA Selenium intake reduces serum C3, an early marker of metabolic syndrome manifestations, in healthy young adults Eur J Clin Nutr 2009 63 858 864 10.1038/ejcn.2008.48 18985060

[B14] Puchau B Zulet MA Hermsdorff HHM Navarro-Blasco I Martínez JA Nail antioxidant trace elements are inversely associated with inflammatory markers in healthy young adults Biol Trace Elem Res 2010 133 304 312 10.1007/s12011-009-8443-5 19582378

[B15] Volp ACP Bressan J Hermsdorff HHM Zulet MA Martínez JA Selenium antioxidant effects and its link with inflammation and metabolic syndrome Rev Nutr 2010 23 581 590 10.1590/S1415-52732010000400009

[B16] Burgos Alves MI Aviles Plaza F Martínez-Tomas R Sánchez-Campillo M Larque E Pérez-Llamas F Martínez Hernández P Parra Pallares S Oxidized LDL and its correlation with lipid profile and oxidative stress biomarkers in young healthy Spanish subjects J Physiol Biochem 2010 66 221 227 10.1007/s13105-010-0028-4 20652473

[B17] Hulten LM Ullstrom C Krettek A van Reyk D Marklund SL Dahlgren C Wiklund O Human macrophages limit oxidation products in low density lipoprotein Lipids Health Dis 2005 4 6 10.1186/1476-511X-4-6 15745457 PMC555960

[B18] Shen G Zhao FZ Zhu F 4P-0964 Impact of oxidized and glycated low-density lipoproteins on reactive oxygen species and glutathione redox system in vascular endothelial cells Atheroscler Suppl 2003 4 286

[B19] Puchau B Zulet MA Urtiaga G Navarro-Blasco I Martínez JA Asymmetric dimethylarginine association with antioxidants intake in healthy young adults: a role as an indicator of metabolic syndrome features Metabolism 2009 58 1483 1488 10.1016/j.metabol.2009.04.037 19586644

[B20] WHO World Health Organization. Obesity: preventing and managing the global epidemic WHO Technical Report Series 894 1998 11234459

[B21] Bressan J Hermsdorff HHM Moreira EAM, Chiarello PG A Epidemia da Obesidade: a causa, o tratamento e o ambiente Atenção Nutricional: Abordagem dietoterápica em adulto Coleção Nutrição e Metabolismo 2008 Rio de Janeiro: Guanabara Koogan 75 94

[B22] Durnin JV Womersley J Body fat assessed from total body density and its estimation from skinfold thickness: measurements on 481 men and women aged from 16 to 72 years Br J Nutr 1974 32 77 97 10.1079/BJN19740060 4843734

[B23] Hermsdorff HHM Puchau B Zulet MA Martínez JA Association of body fat distribution with proinflammatory gene expression in peripheral blood mononuclear cells from young adult subjects OMICS 2010 14 297 307 10.1089/omi.2009.0125 20450441

[B24] Perloff D Grim C Flack J Frohlich ED Hill M McDonald M Morgenstern BZ Human blood pressure determination by sphygmomanometry Circulation 1993 88 2460 2470 8222141 10.1161/01.cir.88.5.2460

[B25] Friedewald WT Levy RI Fredrickson DS Estimation of the concentration of low-density lipoprotein cholesterol in plasma, without use of the preparative ultracentrifuge Clin Chem 1972 18 499 502 4337382

[B26] Castelli WP Cholesterol and lipids in the risk of coronary artery disease--the Framingham Heart Study Can J Cardiol 1988 4 Suppl A 5A 10A 3179802

[B27] Matthews DR Hosker JP Rudenski AS Naylor BA Treacher DF Turner RC Homeostasis model assessment: insulin resistance and beta-cell function from fasting plasma glucose and insulin concentrations in man Diabetologia 1985 28 412 419 10.1007/BF00280883 3899825

[B28] Navarro-Blasco I Alvarez-Galindo JI Selenium content of Spanish infant formulae and human milk: influence of protein matrix, interactions with other trace elements and estimation of dietary intake by infants J Trace Elem Med Biol 2004 17 277 289 10.1016/S0946-672X(04)80030-0 15139390

[B29] Puchau B Zulet MA Hermsdorff HH Navarro-Blasco I Martinez JA Nail antioxidant trace elements are inversely associated with inflammatory markers in healthy young adults Biol Trace Elem Res 2010 133 304 312 10.1007/s12011-009-8443-5 19582378

[B30] Ainsworth BE Haskell WL Whitt MC Irwin ML Swartz AM Strath SJ O'Brien WL Bassett DR Jr Schmitz KH Emplaincourt PO Compendium of physical activities: an update of activity codes and MET intensities Med Sci Sports Exerc 2000 32 S498 504 10.1097/00005768-200009001-00009 10993420

[B31] Martínez-González MA López-Fontana C Varo JJ Sánchez-Villegas A Martínez JA Validation of the Spanish version of the physical activity questionnaire used in the Nurses' Health Study and the Health Professionals' Follow-up Study Public Health Nutr 2005 8 920 927 16277809 10.1079/phn2005745

[B32] Willett WC Nutritional Epidemiology 1998 2 New York: Oxford University Press

[B33] Barbosa KB Bressan J Zulet MA Martínez JA [Influence of dietary intake on plasma biomarkers of oxidative stress in humans] An Sist Sanit Navar 2008 31 259 280 19165292 10.4321/s1137-66272008000500006

[B34] Hermsdorff HHM Zulet MA Puchau B Bressan J Martínez JA Association of retinol-binding protein-4 with dietary selenium intake and other lifestyle features in young healthy women Nutrition 2009 25 392 399 10.1016/j.nut.2008.09.015 19056238

[B35] Zulet MA Puchau B Hermsdorff HHM Navarro C Martínez JA Dietary selenium intake is negatively associated with serum sialic acid and metabolic syndrome features in healthy young adults Nutr Res 2009 29 41 48 19185776 10.1016/j.nutres.2008.11.003

[B36] Dhingra S Bansal MP Hypercholesterolemia and apolipoprotein B expression: regulation by selenium status Lipids Health Dis 2005 4 28 10.1186/1476-511X-4-28 16271152 PMC1291393

[B37] Kaur HD Bansal MP Studies on HDL associated enzymes under experimental hypercholesterolemia: possible modulation on selenium supplementation Lipids Health Dis 2009 8 55 10.1186/1476-511X-8-55 20015371 PMC2805657

[B38] Natella F Fidale M Tubaro F Ursini F Scaccini C Selenium supplementation prevents the increase in atherogenic electronegative LDL (LDL minus) in the postprandial phase Nutr Metab Cardiovasc Dis 2007 17 649 656 10.1016/j.numecd.2006.05.002 17306517

[B39] Behne D Alber D Kyriakopoulos A Long-term selenium supplementation of humans: selenium status and relationships between selenium concentrations in skeletal muscle and indicator materials J Trace Elem Med Biol 2010 24 99 105 10.1016/j.jtemb.2009.12.001 20413067

[B40] Rayman MP Food-chain selenium and human health: emphasis on intake Br J Nutr 2008 100 254 268 18346308 10.1017/S0007114508939830

[B41] Lapointe A Couillard C Piche ME Weisnagel SJ Bergeron J Nadeau A Lemieux S Circulating oxidized LDL is associated with parameters of the metabolic syndrome in postmenopausal women Atherosclerosis 2007 191 362 368 10.1016/j.atherosclerosis.2006.03.036 16677652

[B42] Sjogren P Basu S Rosell M Silveira A de Faire U Vessby B Hamsten A Hellenius ML Fisher RM Measures of oxidized low-density lipoprotein and oxidative stress are not related and not elevated in otherwise healthy men with the metabolic syndrome Arterioscler Thromb Vasc Biol 2005 25 2580 2586 10.1161/01.ATV.0000190675.08857.3d 16224051

[B43] Culleton BF Larson MG Kannel WB Levy D Serum uric acid and risk for cardiovascular disease and death: the Framingham Heart Study Ann Intern Med 1999 131 7 13 10391820 10.7326/0003-4819-131-1-199907060-00003

[B44] Cicoira M Zanolla L Rossi A Golia G Franceschini L Brighetti G Zeni P Zardini P Elevated serum uric acid levels are associated with diastolic dysfunction in patients with dilated cardiomyopathy American heart journal 2002 143 1107 1111 10.1067/mhj.2002.122122 12075270

[B45] Hermsdorff HHM Zulet MA Puchau B Martínez JA Central adiposity rather than total adiposity measurements are specifically involved in the inflammatory status from healthy young adults Inflammation 2011 34 161 170 10.1007/s10753-010-9219-y 20467889

[B46] Holvoet P Kritchevsky SB Tracy RP Mertens A Rubin SM Butler J Goodpaster B Harris TB The metabolic syndrome, circulating oxidized LDL, and risk of myocardial infarction in well-functioning elderly people in the health, aging, and body composition cohort Diabetes 2004 53 1068 1073 10.2337/diabetes.53.4.1068 15047623

[B47] Sigurdardottir V Fagerberg B Hulthe J Circulating oxidized low-density lipoprotein (LDL) is associated with risk factors of the metabolic syndrome and LDL size in clinically healthy 58-year-old men (AIR study) J Intern Med 2002 252 440 447 10.1046/j.1365-2796.2002.01054.x 12528762

