# Supplementary material for: Relationship of oxidized low density lipoprotein with lipid profile and oxidative stress markers in healthy young adults: a translational study
Source: Lipids Health Dis. 2011 Apr 19;10:61. doi: 10.1186/1476-511X-10-61 (PMC3110141; doi:10.1186/1476-511X-10-61)
Supplement: Additional file 1 — Table S1: Spearman bivariate correlation between anthropometric data and ox-LDL concentrations (n = 160). [file 1476-511X-10-61-S1.DOC]

### Additional file 1

### Table S1 Spearman bivariate correlation between anthropometric data and ox-LDL concentrations (n= 160)

| **Anthropometric data** | **Spearman correlations** | |
| --- | --- | --- |
|  | r_S_ | *P*-value |
| Body mass index (kg/m^2^) | 0.13 | 0.095 |
| Waist circumference (cm) | 0.06 | 0.487 |
| Waist-to-hip ratio | 0.03 | 0.689 |
| Sum of 4 skinfold thicknesses (mm) | 0.09 | 0.326 |
| Total body fat (%) | 0.05 | 0.559 |
| Body fat mass (kg) | 0.07 | 0.178 |
